# Positive and Negative Affect and Eating Behavior Among Adults: The Mediating Role of Emotion Regulation

**DOI:** 10.3390/brainsci16010106

**Published:** 2026-01-19

**Authors:** Despoina Kourtidi, Evangelos Ntouros, Agorastos Agorastos

**Affiliations:** 1 School of Medicine, Faculty of Health Sciences, Aristotle University of Thessaloniki, 56430 Thessaloniki, Greece; despina952000@gmail.com; 2 II. University Department Psychiatry, School of Medicine, Faculty of Health Sciences, Aristotle University of Thessaloniki, 56430 Thessaloniki, Greece; durogoo8@gmail.com

**Keywords:** negative affect, positive affect, eating behavior, emotion regulation

## Abstract

**Background**: Emotions substantially influence human eating behavior, but while negative affect has been consistently associated with maladaptive eating patterns, the role of positive affect remains underexplored. Thereby, emotion regulation (ER) is considered a key mechanism through which affective states may influence eating behavior. However, its mediating role remains unclear, particularly among non-clinical populations. **Objectives**: This study aimed to investigate the potential mediating role of ER in the relationship between negative and positive affect and maladaptive eating behavior in a non-clinical adult sample. **Methods**: This cross-sectional online survey was administered to a general-population convenience sample of 189 adults. Participants completed four standardized self-report questionnaires: Eating Attitudes Test (EAT-26), Positive and Negative Affect Schedule (PANAS), Difficulties in Emotion Regulation Scale (DERS-36), and Depression Anxiety Stress Scale (DASS-21). Correlational analyses and multiple regression models were used to examine the relationships between variables and to test the mediating role of ER. **Results**: Negative affect was significantly associated with both maladaptive eating behavior (*r* = 0.29, *p* < 0.01) and ER difficulties (*r* = 0.51, *p* < 0.01). Positive affect was only negatively related to emotion dysregulation (*r* = −0.47, *p* < 0.01). ER did not mediate the relationship between either positive or negative affect and maladaptive eating behavior. **Conclusions**: Findings underscore the influence of negative affect in maladaptive eating behavior, independently of ER. Although positive affect did not directly predict disordered eating behavior, its association with reduced ER difficulties warrants further exploration of its potential protective role.

## 1. Introduction

Emotions are complex post-appraisal responses that involve coordinated environmental, physiological, and psychological influences [1,2,3,4] guiding decision-making and motivating survival-targeted, socially adaptive, and goal-directed behavior by signaling an individual’s needs, values, and intentions to themselves and others [2,3]. Different emotions differentially influence cognition, behavior, and physiological responses [3], and specific ones are often associated with various clinical outcomes, including stress, mood, and anxiety symptoms, as well as disordered eating [5,6,7].

Thereby, theoretical models and previous research propose a valid distinction between negative and positive affect [8,9]. Negative affect refers to a general state of discomfort such as repulsion, anger, disgust, fear, guilt, nervousness, despair, and stress [10,11], while positive affect corresponds to ten representative emotions such as joy, gratitude, serenity, interest, hope, pride, amusement, inspiration, awe, and love [12]. While negative affect has been related to clinical symptoms of depression [5], stress [7], post-traumatic stress [13], somatic problems [14,15], and low self-esteem [14], positive affect is considered to be beneficial to one’s health [10], and has been associated with better stress coping [16] and higher self-esteem [17]. Interestingly, both negative and positive emotions have been differentially linked to eating behavior and related disorders [6,18].

Eating behavior is a broad term that describes one’s way of eating, dieting, and picking foods, and it can also refer to the presence of an eating disorder [19]. Apart from sociocultural parameters, personal body standards, and promoted eating and health behaviors [20,21,22], the current individual’s emotional state constitutes a very important factor influencing eating behavior and even eating speed, chewing, digestion, and metabolism [23]. Macht [24] mentions five pathways through which emotions influence eating: (1) emotional food picking, (2) emotional suppression of food intake, (3) impaired cognitive control, (4) emotional eating, and (5) emotional shaping of eating behavior. Thereby, negative affect is mostly linked to increased food intake [25,26], binge eating [6], and emotional eating [27]. Negative affect often functions as a predictive factor for binge eating episodes [28] and preference for unhealthy food [29]. Other studies associate negative emotions with reduced food intake and specifically sadness, anger, disgust [30], guilt [26], and exhaustion, mostly among women [31]. Previous research emphasizes the association between negative emotions, loss of control, and compensatory behaviors [32].

On the contrary, there is a literature gap with respect to the relation between positive emotions and eating behavior [6,18,24]. Even though some studies support the correlation between high positive affect and increased food intake [6,25,29], other findings highlight that lower positive affect is the most important factor in binge eating [18,33].

In this context, emotion regulation (ER) seems particularly important. ER refers to the process of managing emotional responses and their time or way of expression in order to align with individual or social goals [2,34,35]. The primary characteristic of ER is the activation of an emotion modification goal, influencing the final emotional response [3]. Gross [2] first suggested the “Process Model of ER” that describes five distinct ER strategies: (1) situation selection, (2) situation modification, (3) attentional deployment, (4) cognitive change, and (5) response modulation. Later on, the concept of “appraisal” was additionally incorporated into the “Extended Process Model of ER” [3]. On the other hand, Parkinson and Totterdell [36] recommended the distinction between cognitive and behavioral strategies, while Aldao et al. [34] proposed six different ER strategies: (1) acceptance, (2) problem-solving, (3) reappraisal, (4) avoidance, (5) rumination, and (6) suppression, with the last three being considered maladaptive strategies [37]. Another approach by Hofmann and Kashdan [38] is based on one’s ability to regulate emotions and suggests three determinant ER factors: (1) concealing, (2) adjusting, and (3) tolerating. Finally, other authors also indicate emotional awareness as an important aspect of ER [39,40]. Effective ER has been linked to good health, well-being, and prosperity [34], while maladaptive ER strategies are related to psychopathology and mental disorders [41], such as depression [42], anxiety [37], social anxiety [43], obsessive–compulsive disorder [44], and eating disorders [40,45,46]. Another two very important factors that have been found to influence ER are age and gender, however, with conflicting results in the literature according to age groups and clinical samples investigated [47,48].

With respect to eating disorders, eating behavior is considered a critical ER strategy, while eating problems reflect dysfunctional ER and emotional avoidance [34,40,46]. Ultimately, food often serves as an emotion modification tool [2] and part of a maladaptive ER strategy [39]. Thereby, emotional eating is linked to dysfunctional ER and negative emotions (e.g., sadness) [46], while binge eating is related to positive emotions as well [18]. But even though a dysfunctional ER has been reported in anorexia and bulimia [49,50], its role has also been recognized in the eating behavior of non-clinical populations [39]. Similarly, Markey and Vander Wal’s [45] results, while not confirming the mediating role of ER between negative affect and bulimia, highlighted the significance of emotions in disordered eating. However, there is an evident literature gap regarding the role of positive emotions in clinically significant eating behaviors [51].

Accordingly, the aim of this study is to examine the mediating role of ER between eating behavior and positive and negative emotions in a non-clinical, adult population, as most findings in international literature are reported in clinical samples. Similar findings have been discussed in previous research in relation to childhood trauma [52], emotional abuse [53,54], and depressive symptoms [55], but not directly to negative or positive affect. The present study may provide significant novel clinical information about the influence of positive and negative emotions on eating practices in everyday life and how ER plays a key role in shaping eating habits. The use of a general population sample supports a dimensional approach, capturing variability in affect, emotion regulation, and eating behavior across the full spectrum of severity, including subclinical manifestations.

## 2. Materials and Methods

### 2.1. Study Procedures

This study included a non-probability, general population convenience sample of adults aged 18–65, with no current or history of any axis-I mental disorder and/or intake of psychiatric medication, as per self-declaration by the participants. Participants were mainly recruited by a snowball approach through several channels of targeted/untargeted, personal/institutional advertisement using electronic dissemination platforms (e.g., social media, personal networks, press releases, etc.). Participation was voluntary, and all individuals were informed in advance about the study’s purpose, the potential risks/benefits, and other ethical issues (confidentiality, anonymity, withdrawal at any time). After provision of electronic informed consent, participants were directed to the electronic forms to fill out all questionnaires. Participants completed demographic data and four instruments listed below. The total completion time was estimated at approximately 15–18 min. All data were collected anonymously and stored in an offline PC using appropriate coding.

### 2.2. Measures

- *Eating behavior* was measured with the Eating Attitudes Test, EAT-26 [56], a widely used self-rate screening tool with good reliability and internal consistency, evaluating eating practices and screening for the risk of eating disorders. The measure includes 26 items on a Likert scale from 0 (never) to 4 (always), contributing to three subscales: (1) Dieting, (2) Bulimia and Food Preoccupation, and (3) Oral Control. Higher EAT-26 total and subscale scores indicate more severe maladaptive or disordered eating attitudes and behaviors. Scores above 20 signify a high risk for an eating disorder. The Greek version of the tool has demonstrated satisfactory reliability and is considered suitable for use in the Greek population [57]. In our study, EAT-26 showed good internal consistency for the total scale (Cronbach’s α = 0.84) and its subscales: Dieting (α = 0.81) and Bulimia/Food Preoccupation (α = 0.75), whereas Oral Control demonstrated lower reliability (α = 0.56).
- *Positive and negative emotions* were measured with the Positive and Negative Affect Schedule, PANAS [58], a self-rate instrument measuring emotions the person experienced during the last year on a Likert scale from 1 (very slightly or not at all) to 5 (extremely) with good reliability and internal consistency. It consists of two subscales: negative (10 items) and positive emotions (10 items). The Greek version of PANAS has shown good psychometric properties [59]. In our study, PANAS exhibited good reliability for both positive (α = 0.80) and negative affect (α = 0.86), with the total scale also showing acceptable internal consistency (α = 0.75), allowing its use as a tool to measure emotional intensity.
- *Emotion regulation* was evaluated with the Difficulties in Emotion Regulation Scale, DERS-36 [60], a self-rate tool with very good reliability that includes 36 items, scored on a Likert scale from 1 (almost never) to 5 (almost always). A higher score indicates corresponding ER deficits. DERS-36 consists of six subscales: (1) nonacceptance of emotional responses, (2) difficulties engaging in goal-directed behavior, (3) impulse control difficulties, (4) lack of emotional awareness, (5) limited access to emotion regulation strategies, and (6) lack of emotional clarity. The six-factor structure has been confirmed in the Greek version of the measure [61]. In our study, DERS-36 demonstrated excellent internal consistency (α = 0.91), with its subscales showing acceptable to good internal consistency: Acceptance (α = 0.78), Clarity (α = 0.81), Strategies (α = 0.79), Awareness (α = 0.75), Impulsivity (α = 0.82), and Goals (α = 0.81).
- Stress, anxiety, and depression during the past week were evaluated by the Depression Anxiety Stress Scale, DASS-21 [62]. This widely used self-rate questionnaire consists of 21 items grouped in 3 subscales: (1) Depression, (2) Anxiety, and (3) Stress. Answers are given on a Likert scale from 0 (never) to 3 (almost always). High reliability has been confirmed in the Greek population [63]. In our study, DASS-21 exhibited excellent reliability for the total scale (α = 0.95) and its subscales: Depression (α = 0.89), Anxiety (α = 0.90), and Stress (α = 0.89).

### 2.3. Statistical Analysis

For DERS-36 and EAT-26, reverse-scored items were identified. All scales were computed according to instructions, the Kolmogorov–Smirnov test was used to assess normality, and then internal consistency was assessed. Internal consistency of all scales and subscales was assessed using Cronbach’s alpha coefficient. Cronbach’s alpha values were interpreted according to conventional guidelines, with α ≥ 0.70 indicating acceptable, α ≥ 0.80 good, and α ≥ 0.90 excellent internal consistency. *T*-tests were conducted, where appropriate, to assess group differences. One-way ANOVAs were conducted to examine age differences across all variables and their subscales. For ANOVA, age was categorized into three groups: 18–30 (n = 77), 31–50 (n = 61), and 51+ (n = 51). Psychometric properties of each scale were calculated, and descriptive statistics were used to examine scale and subscale scores. Pearson’s correlation was employed to assess linear associations. Effect sizes for correlations were interpreted using Cohen’s conventional benchmarks (small ≈ 0.10, moderate ≈ 0.30, large ≈ 0.50), while acknowledging that effect magnitude should be interpreted in context. Regression analyses were performed to determine the total effect of independent variables on dependent variables and direct effects. The Sobel test was used to examine the potential mediating role of ER and each ER subscale in the relationship between negative/positive affect and maladaptive eating behavior. Analyses were conducted using IBM SPSS Statistics 29. All tests of significance were 2-tailed, and *p*-values < 0.05 were considered statistically significant.

## 3. Results

### 3.1. Participants

The sample consisted of 189 adults (140 ♀, 74%) with an approximate mean age of 38.83 ± 14.31. Of them, 84.6% had at least a university degree or higher, while approx. 26% declared being single, 31% in a relationship, and 43% married.

### 3.2. Descriptive Statistics

For maladaptive eating behavior, only 25 out of 189 participants (12.7%) scored higher than 20 on the total EAT-26 score. A higher score was observed in the subscale of Dieting (6.03 ± 5.96), compared to Bulimia (1.9 ± 2.82) and Oral Control (2.35 ± 2.69). For emotions, higher scores were observed for positive (34.84 ± 5.94), rather than negative emotions (24.57 ± 7.39). For ER, participants scored 92.58 ± 19.26 (moderate to high experience of ER difficulties). The majority of ER deficits were related to limited access to effective ER strategies (23.46 ± 5.79), while the lowest score was observed for emotional clarity (10.67 ± 3.78). The mean score for depression-anxiety-stress was 17.17 ± 14.30, indicating a general tendency toward mild symptomatology. However, 62 (32.8%) of all participants scored 20 or above, reflecting moderate symptoms, of whom 20 (10.6%) scored 40 or above, indicating severe symptoms. The subscale with the highest score was Stress (6.90 ± 4.95), followed by Depression (5.46 ± 5.19), and Anxiety (4.82 ± 5.25). Reliability and internal consistency values of questionnaires and subscales have been presented in the Methods section.

Independent samples *t*-tests revealed that women scored significantly higher than men in PANAS-negative emotions (*p* = 0.017), ER difficulties (*p* = 0.026), and DAS total score (*p* = 0.016). Specifically, results revealed gender differences in four subscales: ER-Acceptance (*p* = 0.007), ER-Strategies (*p* < 0.001), ER-Goals (*p* = 0.023), and DAS-Depression (*p* < 0.001) (Table 1). ANOVAs investigating psychometric differences in all questionnaires and subscales between the three different age groups 18–30 (n = 77), 31–50 (n = 61) and 51+ (n = 51) revealed significant age differences only in three subscales: Eating Behavior-Bulimia (*p* = 0.007), ER-Clarity (*p* = 0.002) and ER-Goals (*p* = 0.039). Post hoc comparisons (Bonferroni) revealed that participants aged 31–50 scored significantly higher (M = 2.68) in Bulimia than those aged 51+ (M = 1.01). For Clarity, participants aged 18–30 scored significantly higher (M = 11.83) than both 31–50 (M = 9.7) and 51+ (M = 10.05) groups. For Goals, post hoc comparisons did not reveal significant differences.

### 3.3. Correlations

#### 3.3.1. Emotions and Eating Behavior

Negative affect was significantly and positively correlated with maladaptive eating behavior with a moderate effect size (*r* = 0.29, *p* < 0.01, Table 2). The subscales with the highest positive correlation were Dieting (*r* = 0.29, *p* < 0.01) and Bulimia (*r* = 0.23, *p* < 0.01), both with moderate effect size, while Oral Control was not significantly correlated with negative affect (*r* = 0.09) (Table 3). On the other hand, positive affect did not show any statistically relevant positive or negative correlation to maladaptive eating, nor was it significantly correlated with any of the EAT subscales. Lastly, our results revealed a positive and statistically significant association between maladaptive eating behavior and emotion intensity (PANAS total score) with a moderate effect size (*r* = 0.24, *p* < 0.01). Specifically, emotion intensity was associated with a moderate effect size with Dieting (*r* = 0.22, *p* < 0.01) and with a small to moderate effect size with Bulimia (*r* = 0.17, *p* < 0.05). Oral Control was once again not significantly correlated with emotion intensity.

#### 3.3.2. ER and Emotions

Results revealed that negative emotions are positively correlated with ER difficulties with a large effect size (*r* = 0.51, *p* < 0.01), while positive emotions are also negatively correlated with a large effect size (*r* = −0.47, *p* < 0.01). Awareness was the only ER subscale with no significant correlation with negative affect. Table 4 shows correlations between emotions and the DERS subscales.

#### 3.3.3. ER and Eating Behavior

Results showed a positive correlation between ER deficits and disordered eating with a small effect size (*r* = 0.16, *p* < 0.05). Nonacceptance was the only ER subscale to be related to all EAT subscales: Dieting (*r* = 0.15, *p* < 0.05), Bulimia (*r* = 0.18, *p* < 0.05), and Oral Control (*r* = 0.15, *p* < 0.05) with small effect sizes, while Strategies and Impulsivity demonstrated significant associations as well. Awareness was not correlated with any EAT subscale (Table 4).

#### 3.3.4. Depression, Anxiety, Stress, and Eating Behavior

There was a positive correlation between depression-anxiety-stress and maladaptive eating with a moderate effect size (*r* = 0.26, *p* < 0.01). Specifically, eating problems were associated with depression (*r* = 0.25, *p* < 0.01), anxiety (*r* = 0.24, *p* < 0.01), and stress (*r* = 0.23, *p* < 0.01), all with moderate effect sizes. Dieting (*r* = 0.24, *p* < 0.01) and Bulimia (*r* = 0.29, *p* < 0.01) were positively correlated with DASS-21, both with moderate effect sizes. Oral Control was not significantly related to any DASS-21 subscale (Table 5).

#### 3.3.5. Gender Differences

Fisher’s tests were conducted to compare the correlations between genders. The only significant difference emerged for the correlation between eating behavior and ER (*z* = −1.82, *p* = 0.034). Among women, eating problems and ER difficulties were positively correlated with a small to moderate effect size (*r* = 0.22, *p* < 0.05), whereas among men the correlation was negative and not statistically significant (*r* = −0.09, *p* > 0.05). The correlations among all variables for both genders are presented in Table 6.

### 3.4. Mediating Role of ER Between Emotions and Eating Behavior

**Figure 1:**
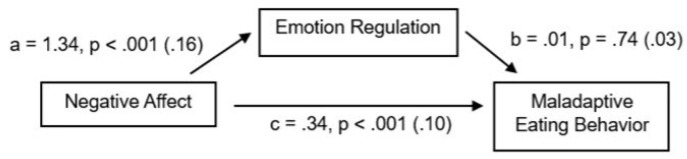
Direct effect (unstandardized B, *p*-values, and SE): the mediating role of emotion regulation between negative affect and eating behavior.

When it comes to negative affect, the regression analysis showed that the overall effect of negative emotions on maladaptive eating behavior was statistically significant (*Β* = 0.36, *p* < 0.001), with approximately 8.4% of the variance in eating behavior explained by negative affect. The direct effects were also calculated: (1) negative effect on ER (*Β* = 1.34, *p* < 0.001), (2) ER on eating behavior (*Β* = 0.01, *p* = 0.74), and (3) negative effect on eating behavior (*Β* = 0.34, *p* < 0.001). Thus, only the direct effect of ER on disordered eating was not statistically significant. The Sobel test was also conducted, yielding *p* = 0.66, which exceeds the 0.05 significance threshold. Therefore, the indirect effect is not statistically significant, and there is no mediation role of ER between negative affect and disordered eating. So, the effect of negative emotion on maladaptive eating behavior is direct and independent of ER and its subscales (Figure 1).

On the other hand, regression analysis showed no overall effect of positive affect on eating behavior (*Β* = −0.008, *p* = 0.94). Therefore, no further analyses could be conducted, and thus, there is no mediation of ER between positive affect and eating behavior. Lastly, we examined the potential mediation of ER in the relationship between maladaptive eating behavior and emotions (total score), and even though the regression analysis showed a significant overall effect (*Β* = 0.25, *p* < 0.001), further tests indicated no direct effect of emotions on ER (*p* = 0.11). Thus, emotion intensity is directly—not indirectly through ER—associated with disordered eating.

## 4. Discussion

The present study examined the relationship between negative and positive emotions and maladaptive eating behavior in a non-clinical adult sample while assessing the mediating role of ER in this relationship. The main findings were as follows: (1) negative affect was linked to disordered eating tendencies with a moderate effect size, (2) negative affect was positively correlated to ER difficulties, while positive affect was negatively correlated, both with a large effect size, and (3) ER did not mediate the relationship between affect and maladaptive eating patterns.

### 4.1. Emotions and Eating Behavior

Negative and positive emotions appear to function as two distinct variables, representing opposite ends of the same continuum [64]. Thus, eating as a response to negative or positive emotions reflects two different processes [65]. Supporting this claim, the results of the present study revealed that negative and positive affect are negatively related, and therefore, one’s high negative affect indicates lower positive affect and vice versa.

Analysis supported previous research that associates negative affect with disordered eating [6,26,29,30,66], with the present findings indicating statistically significant but small-to-moderate associations. Specifically, negative showed small-to-moderate positive correlations with Dieting and Bulimia, but not Oral Control. Such findings are supported by the existing literature, as negative affect has been linked to strict diets and the aspiration “to be thin” [67] or with increased food intake [6]. On the contrary, Oral Control is mostly linked to obese people [68,69] or severe binge-eating episodes [70], whereas participants in this study constituted a non-clinical sample, as reflected in their eating behavior scores.

Regarding positive emotions, the existing literature provides contradictory findings [29,65] that highlight the need for further research. The present study did not confirm a correlation between positive affect and eating problems, suggesting that any potential relationship, if present, is likely small in magnitude. Such findings may reflect that prior research has primarily examined the lack of positive affect, rather than its intensity [18]. Thus, our results align more with other findings that indicate a negative relationship between positive affect and eating psychopathology [71]. Notably, emotional intensity was found to be related to maladaptive eating practices, indicating that experiencing high positive or negative emotions may influence eating patterns. Overall, however, although several associations reached statistical significance, their effect sizes were generally small to moderate, indicating reliable but modest relationships.

### 4.2. ER and Emotions

Emotion dysregulation has been linked to negative [72] and positive emotions [51], although our results confirmed a positive correlation with a large effect size only for negative emotions in the current non-clinical sample. First, higher negative affect was revealed to indicate more ER problems, and prior research has indeed concluded that strategies such as denial, rumination [34,41], and suppression [2] are connected to negative emotions and psychopathology. Second, higher positive affect was revealed to indicate fewer ER problems. In line with this finding, previous studies support that positive emotions are linked to adaptive ER strategies, such as reappraisal [73], acceptance, and problem-solving [34].

Contradictory findings highlight the need for further research, and especially the examination of dysregulation of positive emotion as a distinct variable [73]. The present study employed DERS-36, which primarily focuses on negative ER [74] and is therefore more sensitive to fluctuations in negative emotions.

### 4.3. ER and Eating Behavior

Disordered eating is considered to be a maladaptive ER strategy [34,39], and the findings of the present study are partially consistent with this perspective, showing small but statistically significant associations. Applying the results to Hofmann and Kashdan’s model [38], we suggest that disordered eating practices are linked to one’s inability to regulate emotions, not to specific strategies. In other words, food may function as “concealing” [38], one’s way to suppress emotions [34].

According to Gross [2,3], though, problematic eating behavior may emerge as a result of maladaptive ER strategies. One’s appraisal that an emotional state needs to be regulated may result in using food as a modification tool. Indeed, our results revealed that Dieting and Bulimia are related to limited access to ER strategies. Nonacceptance of emotional responses was also related to Dieting and Bulimia, as other studies indicate [39,75], although these associations were small in magnitude. Oral Control was not significantly related to ER, possibly because this subscale has not been widely linked to non-clinical populations [69].

We conclude that difficulties in (1) acceptance of emotional responses and (2) limited access to ER strategies were most closely related to disordered eating patterns. Similarly, (1) Dieting and (2) Bulimia emerged as the EAT subscales most strongly associated with ER difficulties. Thus, while emotion regulation difficulties appear relevant, the observed effects suggest a small to modest contribution rather than a strong determinant of eating behavior.

### 4.4. Mediating Role of ER

A key implication of the current study concerned the potential mediating role of ER in the association between negative and positive affect and maladaptive eating behavior. Nevertheless, this mediation was not confirmed. Our findings suggest that negative affect is directly but modestly associated with maladaptive eating behavior, independent of ER. This means that higher levels of negative affect are associated with an increased likelihood of problematic eating behavior, although the magnitude of this association is limited.

So, why was the mediating role of ER not supported in this study, while other findings suggest so [52,53,54]? First, the present study used PANAS, which measures emotions the person experienced during the last year, while other studies employed measures of more chronic emotional states, such as depression, emotional abuse, or trauma [40,52,55]. Second, this study did not examine the factor of “emotional eating,” which is considered to be heavily influenced by emotional changes [40]. Lastly, as our sample was non-clinical, future studies could examine whether ER mediates the relationship between affect and eating behavior in clinical populations.

### 4.5. Gender Differences

Our findings revealed significant gender differences in emotional burden in general, with women reporting higher levels of negative affect, emotion dysregulation, and depression-anxiety-stress. However, contrary to prior research that has linked women to strict diets [21], binge-eating episodes [6], and general eating problems [31], no gender differences emerged in disordered eating. This result may arise from the size difference (ΝM = 49 and ΝW = 140) or may indicate that gender differences regarding eating behavior tend to decrease, as men are progressively encountering food-related problems [76,77].

### 4.6. Limitations and Future Directions

Our study examined mediation using regression-based product-of-coefficients testing. Although bootstrap confidence intervals are often recommended for indirect effects, the present model used observed total scores in a simple single-mediator framework, and conclusions were driven by the near-zero association between the mediator and outcome. Future studies may apply bootstrap-based mediation (e.g., PROCESS or SEM frameworks) and larger samples to increase sensitivity for small indirect effects. Although indirect effects are statistically more demanding to detect, the non-significant direct association between emotion regulation and eating behavior suggests that the absence of mediation is not solely a function of sample size. Future studies with larger samples may further clarify whether emotion regulation plays a mediating role under different conditions or in clinical populations. A larger sample also could enhance the representativeness of the general population, while a more balanced gender distribution could yield more reliable conclusions. Moreover, given the use of DERS-36 and its focus on negative affect and its regulation, future research could incorporate measures assessing positive ER [51] and include clinical samples to provide a more comprehensive understanding of emotions and the mechanisms through which they influence eating behavior.

Our findings in a non-clinical sample indicate that associations between positive and negative affect and eating behavior are present at subclinical levels, supporting a dimensional view of eating-related difficulties. This underscores the importance of examining these relationships in clinical populations and from a developmental perspective to clarify how affective influences on eating behavior may evolve across the lifespan. Finally, it is important to distinguish statistical significance from practical or clinical relevance, as the majority of observed correlations, although consistent and theoretically meaningful, were of small to moderate magnitude

## 5. Conclusions

This study examined the relationship between negative and positive affect and maladaptive eating behavior in a non-clinical adult sample while assessing the mediating role of ER in this relationship. Our results highlight the role of negative emotions in maladaptive eating behavior regardless of ER. ER may be linked to eating behavior, but it appears to act as a reinforcing factor in the influence of negative emotions. Positive emotions, on the other hand, may not have been directly related to disordered eating, but their negative relationship with ER difficulties shows that their role needs to be further investigated. Clinically, these findings highlight negative affect as a potential early target for the prevention of maladaptive eating behaviors, even at subclinical levels. Addressing emotional distress in clinical and preventive interventions may help reduce vulnerability to the development of eating pathology.

## Figures and Tables

**Table 1:** Psychometric scores across all questionnaires and subscales in the total sample (Ν = 189) and across genders.

Variables	Total		Males		Females		*p* Value
	Mean	SD	Mean	SD	Mean	SD	
**Eating behavior**	10.29	9.20	8.38	6.96	10.95	9.80	0.093
Dieting	6.03	5.96	4.67	4.27	6.50	6.39	0.064
Bulimia/Food Preoccupation	1.9	2.82	1.55	2.18	2.02	3.01	0.309
Oral Control	2.35	2.69	2.16	2.53	2.42	2.75	0.550
**Negative emotions**	24.58	7.39	22.40	6.92	25.33	7.42	0.017 *
**Positive emotions**	34.84	5.94	35.77	4.75	34.51	6.28	0.193
**Emotion regulation**	92.57	19.26	87.32	19.09	94.41	19.04	0.026 *
Nonacceptance	15.01	4.76	13.40	4.83	15.57	4.62	0.007 *
Clarity	10.67	3.78	10.48	3.31	10.72	3.94	0.705
Strategies	23.46	5.79	21.14	5.67	24.26	5.63	<0.001 *
Awareness	13.38	4.04	14.22	3.63	13.07	4.15	0.088
Impulsivity	15.37	4.64	14.48	5.13	15.67	4.43	0.156
Goals	14.70	4.05	13.57	3.97	15.10	4.02	0.023 *
**Depression, anxiety, stress**	17.17	14.30	13.36	11.75	18.51	14.89	0.016 *
Depression	5.46	5.19	3.73	3.55	6.06	5.53	<0.001 *
Anxiety	4.82	5.25	3.59	4.29	5.25	5.49	0.057
Stress	6.90	4.95	6.04	4.64	7.20	5.03	0.145

Data are given in means and standard deviations (SD) across questionnaires and subscales. * *p* < 0.05.

**Table 2:** Pearson correlations (r) among all psychometric variables in the total sample (Ν = 189).

Variables	1	2	3	4	5
1. Maladaptive eating behavior	-				
2. Negative affect	0.29 **	-			
3. Positive affect	−0.005	−0.14 *	-		
4. Emotion regulation	0.16 *	0.51 **	−0.47 **	-	
5. Depression, anxiety, stress	0.26 **	0.54 **	−0.28 **	0.58 **	-

** *p* < 0.01, * *p* < 0.05.

**Table 3:** Pearson correlations (r) between emotions and eating behavior in the total sample (Ν = 189).

EAT-26 Subscales	Negative Emotions	Positive Emotions	Emotions
1. Dieting	0.29 **	−0.02	0.22 **
2. Bulimia/Food Preoccupation	0.23 **	−0.02	0.17 *
3. Oral Control	0.09	0.07	0.13

** *p* < 0.01, * *p* < 0.05.

**Table 4:** Pearson correlations (r) between DERS-36 and EAT-26 and PANAS subscales in the total sample (Ν = 189).

DERS-36 Subscales	Dieting	Bulimia/ Food Preoccupation	Oral Control	Negative Emotions	Positive Emotions
1. Nonacceptance	0.15 *	0.18 *	0.15 *	0.55 **	−0.25 **
2. Clarity	0.09	0.17 *	−0.00	0.20 **	−0.46 **
3. Strategies	0.20 **	0.18 **	−0.02	0.59 **	−0.32 **
4. Awareness	−0.01	0.02	−0.04	−0.03	−0.45 **
5. Impulsivity	0.07	0.20 **	−0.17 *	0.38 **	−0.28 **
6. Goals	0.16 *	0.11	0.05	0.34 **	−0.25 **

** *p* < 0.01, and * *p* < 0.05.

**Table 5:** Pearson correlations between DASS-21 and EAT-26 subscales in the total sample (Ν = 189).

Subscales	Dieting	Bulimia/Food Preoccupation	Oral Control
1. Depression	0.23 **	0.27 **	0.06
2. Anxiety	0.21 **	0.38 **	0.06
3. Stress	0.22 **	0.27 **	0.01

** *p* < 0.01.

**Table 6:** Pearson correlations (r) among all psychometric variables for men (Ν = 49).

Variables	1 Men/Women	2 Men/Women	3 Men/Women	4 Men/Women
1. Eating behavior	-			
2. Negative emotions	0.205/0.293 **	-		
3. Positive emotions	0.130/−0.016	0.000/−0.165	-	
4. Emotion regulation	−0.092/0.215 *	0.503 **/0.501 **	−0.365 **/−0.493 **	-
5. Depression, anxiety, stress	0.070/0.284 **	0.588 **/0.518 **	−0.202/−0.294 **	0.574 **/0.576 **

** *p* < 0.01, * *p* < 0.05.

## Data Availability

The data presented in this study are available on request from the corresponding author due to university procedures.

## Abbreviations

The following abbreviations are used in this manuscript:

DASS-21	Depression Anxiety Stress Scale
DERS-36	Difficulties in Emotion Regulation Scale
EAT-26	Eating Attitudes Test
ER	Emotion regulation
PANAS	Positive and Negative Affect Schedule
